# Research on Robot Fuzzy Neural Network Motion System Based on Artificial Intelligence

**DOI:** 10.1155/2022/4347772

**Published:** 2022-02-09

**Authors:** Jie Hu

**Affiliations:** Suzhou University, Institute of Physical Education, Suzhou 234000, Anhui, China

## Abstract

An intelligent controller based on a self-learning interval type-II fuzzy neural network is proposed to make the motion controller of the industrial intelligent robot with good adaptability. This controller has a parallel structure and contains an interval type-II fuzzy neural network and a conventional PD controller. For the design of the interval type-II fuzzy neural network, the interval type-II fuzzy set is established using the slave design method. In the design process of the interval type-II fuzzy set of the front piece, a dual sequence symmetric trapezoidal subordinate function arrangement method is proposed, which makes the self-learning law and stability analysis of the system in an analytic form and facilitates the implementation of the algorithm in hardware. In the design of the neural network self-learning law, a parametric self-learning algorithm based on sliding mode control theory is established to adjust the structural parameters of the interval type-II fuzzy neural network online, and the stability of the system is proved by using Lyapunov's stability theorem. Three sets of validation simulation experiments are given in conjunction with the trajectory tracking problem of the Delta parallel robot. The simulation results show that, in the presence of system uncertainty, the intelligent controller based on interval self-learning interval type-II fuzzy neural network can significantly improve the trajectory tracking accuracy and robustness of the system and make the control system highly adaptable to the environment. Experiments of intelligent control system based on self-learning interval type-II fuzzy neural network and experiments of reusable particle swarm optimal motion planning method are designed, and the effectiveness of the intelligent control system and motion planning method is verified on the experimental platform. The experimental results show that the intelligent control system based on the self-learning interval type-II fuzzy neural network can effectively improve the accuracy and stability of robot trajectory tracking control, and the reusable particle swarm optimal motion planning method can quickly solve the robot motion planning problem with complex constraints online.

## 1. Introduction

Artificial intelligence is both the background of the times and a prerequisite for machines to have intelligence and sociality. Existing studies on human-machine social interaction only consider the shallow expression of human-human social or human-machine interaction, without considering the trends and design requirements of the deeper changes in the human-machine relationship in the upcoming AI era. In the past, machines were used as tools or mediums for human-human interaction, but with the further enhancement of machine intelligence, direct human-machine interaction has gradually evolved [1]. Through the increasingly in-depth human-machine interaction, intelligent machines have functional, emotional, and social effects on people, which leads to the new topic of human-machine social interaction in the era of artificial intelligence [2]. In the context of the new social environment and the development of new human-machine social interaction, we start from the background and the current situation of the study, use literature review and typical example analysis in the early stage of the study to understand the current research process of artificial intelligence, social interaction, and human-machine interaction, explore the part that can be further developed based on the shortage of existing research, and propose the purpose and significance of this study. When IT2FNN is connected in parallel to both ends of the PD controller, the self-learning process of IT2FNN's structural parameters begins. After the learning time of about 1.7 seconds is over, the output signal of IT2FNN replaces the PD controller as the main output source of the control signal. The result is the same. The above simulation results clearly show the influence of the learning process of SLIT2FNN on the output of the controller and the improvement of control accuracy after adopting the parallel intelligent control structure in the second working cycle.

In practical applications, one of the most frequently encountered situations for industrial intelligent robots is trajectory tracking control [3]. If a satisfactory control effect is to be obtained for an industrial robot, there are many problems to be solved in the design of the control system, such as the nonlinearity of the robot body structure, the uncertainty of modeled and unmodeled dynamics, and the diversity of operating conditions, in addition to its dynamics optimization design. In the last decade, many related research works have been done by scholars on the trajectory tracking control problem of parallel robots. Some of them are based on traditional control methods for trajectory tracking control problems: some studies [4, 5] are based on feedback linearization control methods; others [5, 6] are based on adaptive theory, in addition to studies based on variable structure control theory. However, most of the controllers based on traditional control methods need to provide an accurate mathematical model of the controlled object to obtain satisfactory control results [7]. However, obtaining a complete dynamical model of a parallel robot is very difficult and sometimes impossible. Moreover, even if accurate robot system parameters can be obtained experimentally, it is equally difficult to derive a dynamic model of the robot based on these parameters, especially for some parallel robot systems with complex motion mechanisms.

Taking the Delta parallel robot as the object, the forward and inverse kinematics models of the robot are established, and the dynamics model of the robot is established by using the virtual work principal method. On this basis, a robot body mechanism parameter optimization method oriented to the control task requirements is proposed. Firstly, the mechanism parameter optimization algorithm considering anisotropic dynamics characteristics is established, and the optimization models are built with the full-domain drive joint torque index and the full-domain drive joint torque fluctuation index, respectively, and the optimization problems are solved to verify the effectiveness of the algorithm. Then a multiobjective dynamics optimization model based on the full-domain performance index is established, and the multiobjective optimization problem is solved using a multiobjective genetic algorithm, and the computational efficiency and effectiveness of the algorithm are verified. The above model and optimization algorithm of the Delta parallel robot are established using the idea of building blocks to make the robot system simulation platform universal. A parallel intelligent control system based on a self-learning interval type-II fuzzy neural network is proposed and used to solve the trajectory tracking control problem of the Delta parallel robot. This controller is a parallel structure: it contains an interval type-II fuzzy neural network and a conventional PD controller. The IT2FS is designed using a subordinate design approach, based on which a dual sequence symmetric trapezoidal subordinate function arrangement is proposed to give an analytic form to the interval type-II subordinate function for designing the self-learning law and stability analysis. In the design of the neural network self-learning law, a parametric self-learning algorithm based on the SMC theory is proposed to adjust the structural parameters of the interval type-two fuzzy neural network online. The stability of the proposed intelligent control system is proved using Lyapunov's stability theorem. The performance of the established intelligent controller is verified through simulation experiments.

## 2. Related Work

In terms of isotropic optimization problems in the full working domain, Narayan et al. proposed a multiobjective optimization method for the design of structural parameters of a four-degree-of-freedom parallel robot for the robot isotropic design problem with task requirements, using robot sensitivity analysis and standardized workspace volume as objective functions [8]. Rao et al. studied the scale synthesis problem of the Hexaslides robot and gave a method to select the structural parameters of the Hexaslides robot for the machining field [9]. Mirzaey et al. investigated the kinematic optimization problem of a two-degree-of-freedom parallel robot arm using a stepwise optimization method. The number of design variables was reduced by optimizing the parameter relations with an analytic form in the first step [10]. The second step reduces the optimization variables of the optimization problem to one utilizing the given global optimization index and thus solves the kinematic optimization problem of a parallel robotic arm [11]. Li et al. proposed a dynamic isotropic optimization method for the full working domain of a parallel robot, giving a dynamic optimization objective function based on the robot inertia matrix, and demonstrated the validity of its objective function through an optimal design problem of a three-degree-of-freedom tandem robot and a parallel robot [12].

Since we need to obtain a controller with high nonlinear characteristics and smoother control performance, the IT2FLC obtained by the two-terminal fuzzy method is the structure of the type 2 fuzzy logic controller that best meets the expectations of our control system characteristics. Vision servo is based on the visual information provided by the camera to control the robot. Depending on the feedback error used to calculate the control law, the vision servo is divided into position-based, image-based, and motion-based control systems [13]. In the position-based control system, the feedback error is calculated in a three-dimensional Cartesian coordinate space; in the image-based control system, the feedback error is calculated only from the different pixel points of the current image and the reference image; both systems are model based and require rigorous calibration of the camera to determine the exact geometric relationships between the camera and the robot's end-effector and then use these geometric relationships to analyze the feature points in the image [14]. These geometric relationships are then used to analyze the feature points in the image, which are usually selected by humans. In contrast, motion-based control systems are model-free and can calculate feedback errors without any a priori using of frame difference methods, optical flow methods, etc. With the development of technologies such as deep learning and the advent of the era of artificial intelligence, the connection between machines and people is getting closer and closer, and artificial intelligence with human-machine dialogue function is gradually social role-playing, and the human-machine relationship is changing accordingly [15]. The research finds that existing studies on social interaction only consider human-human socialization and do not consider the changes in the human-machine relationship in the coming AI era [16]. To verify whether the motion mode of the real machine is consistent with the motion gait based on the Hop oscillation model proposed in this paper, the wake gait motion of the robot dog can be obtained through the video screenshots during the experiment. This study explores the changes in human-robot social relationships, the deep-seated needs, and ultimate purpose of human-robot social interaction and analyzes how evolving human-like intelligent robots can meet the dynamic deep-seated emotional needs of humans to adapt to human needs.

The communication between man and machine will rise from mechanical external interaction to an emotional level. All specific operational devices will be naturally integrated into the overall information infrastructure. In their place, there will be a wide variety of ubiquitous sensors. The integration of AI, cloud computing, and increasingly intelligent machines will generate data for use in human-computer interaction systems. The study of social interaction has the value of studying and predicting future trends in the evolution of human-machine relationships. Machines gradually take on the attributes of social roles and, on the one hand, have a social connection with people. From another perspective, the advancement of human-like intelligence such as artificial intelligence, which is mainly driven technology, has replaced some of the jobs originally performed by humans, thus causing an identity crisis and technological anxiety in human values. The development of artificial intelligence and human-like emotions, which are mainly emotionally dependent, will have a more profound impact on human society, especially in terms of human identity and emotional dependence in social relations.

## 3. Fuzzy Neural Network Motion System for Robot with Artificial Intelligence

### 3.1. Robot Motion System Design

To realize the robot from the virtual prototype model to the real model, the first step is to draw the vector drawing for the real model and to mark the drawing size on the vector drawing according to the size of the virtual prototype; the common software to draw the structure drawing is Autodesk Computer-Aided Design (AutoCAD) and CorelDRAW. In this paper, we chose AutoCAD as the drawing tool.

The body structure of the robot is three-dimensional. In this paper, the length, width, and depth of each part need to be marked in the drawing design so that the robot body can be assembled by printing out the parts with a three-dimensional structure [17]. The body structure of the quadruped robot was designed concerning the mortise and tenon structure of ancient Chinese architecture, and the torso of the robot was designed to be fixed without screws, but only by inserting the mortise and tenon structure. After the design of the drawing, the drawing was processed by laser engraving, and then the dimensions of the drawing were verified by assembling, and the unreasonable structural parts were modified.

**Figure 1:**
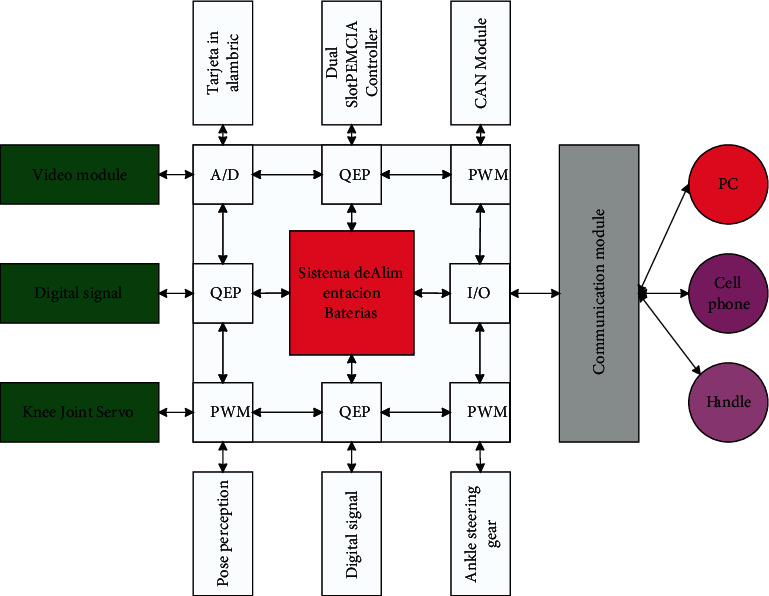
Hardware structure of the robot.

The robot's control method is divided into two types: external command control and automatic adjustment control of the robot's decision system [18]. In external command control, the robot needs to have a signal receiver that can communicate with the outside, and in decision control, the robot needs to be able to sense the surrounding road conditions, audio, and video signals, etc. After receiving the signals, the robot's brain needs to perform a series of operations, such as data calculation and task scheduling, and then control the leg servos to make corresponding movements. The SLIT2FNN controller is in the environment dynamics learning process. The neural network adjusts its own structural parameter values through the parameter self-learning algorithm. At this time, the control signal is mainly provided by the PD controller in the parallel control structure. After the neural network self-learning process is over, IT2FNN replaced the traditional PD controller as the main control signal output in the parallel control structure. At the same time, it can be seen from the figure that the tracking accuracy of the robot has been significantly improved. Therefore, the hardware structure of a robot is like the von Neumann architecture of a computer, which consists of five major parts, each of which cooperates to realize the robot's functions. The robot hardware architecture is shown in Figure 1, where the communication module, the video perception module, and the pose perception module are the input devices of the robot, the servo controlled by PWM (pulse width modulation) signal is the output device of the robot, and the middle part is the operator and controller of the robot.

The robot system framework is a typical hierarchical multiloop control structure, in which the robot system is divided into different layers from top to bottom, and each layer will have multiple tasks to be executed collaboratively. The first three layers are embedded inside the robot, and the last layer is the interaction layer between external users and the robot.

**Figure 2:**
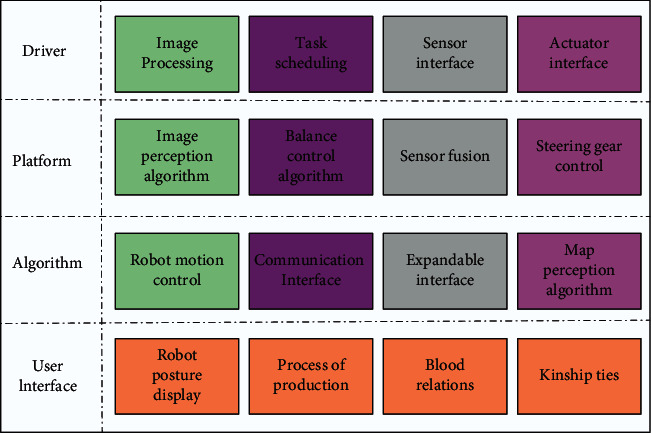
Hierarchical circuit diagram of the robot system.

The main function of the driver layer is to realize the cooperation between the robot hardware and software. The sensor interface is responsible for receiving digital signals from the sensors installed on the robot body, such as receiving video stream signals from the camera, while the main command of the actuator interface is to send commands to the actuator, sending the system control signals to the actuator and controlling the robot's servo to complete the motion function [19]. Firstly, the mechanism parameter optimization algorithm considering the anisotropic dynamic characteristics is established, and the optimization model is established with the global driving joint torque index and the global driving joint torque fluctuation index, respectively, and the optimization problem is solved to verify the effectiveness of the algorithm. The main command of the actuator interface is to send commands to the actuator, send the system control signals to the actuator, control the robot's servo to complete the motion function, and receive the feedback signals from the actuator to sense whether the actual motion of the robot is the same as the control command. The second layer of the multitasking platform layer mainly implements the fusion of sensor data, control of multiple servos, image processing, and task scheduling functions, and the platform layer requires the driver layer and the algorithm layer together to complete the task. The robot system needs to fuse these sensor data and then realize the processing of different sensor data through the corresponding algorithms in the upper algorithm layer. The servo control interface mainly sends the control signal of the servo to the actuator and realizes the control of a certain servo or the parallel control of the whole-body servo according to the task, and the image processing interface accepts the video stream data sent by the camera sensor, and then the image processing interface of the algorithm layer performs calculations, as shown in Figure 2.

The specific manifestation of social relations is the human-computer relationship. Human-computer relations place more emphasis on the individual characteristics of people who influence each other, while social relations refer to the common aspects it contains. Social relations refer to the interpersonal relations formed by people in the process of production and living together. Social relations are mainly divided into blood relations, georelations, and karma relations. Blood relations are rooted in or kinship ties and are the first social relations formed by human beings. They constitute social groups such as families and households. Geo-relationships are based on the spatial and geographical relationships of people. Karmic relations are formed based on the extensive social division of labor and promote social development. Types of interpersonal relations refer to the distinction of types of interpersonal relations. According to Thibault's view of interpersonal interdependence, he classifies interpersonal relationships as minimal interdependence, asymmetric interdependence, reactive interdependence, and mutual interdependence. Minimal interdependence means that the interacting parties act according to their intentions. Asymmetric dependence means that one party's actions are based on the actions of the other party, but with opposite intentions. Reactive interdependence means that one party acts in full accordance with the other party's intentions. Mutual dependence is the most equal and stable form of interaction; e.g., it can be classified as emotional or instrumental interpersonal relationships according to needs.

In terms of the relationship and process of human-computer interaction, the user (human) is constantly changing during the use of the machine. Human behavior and emotions are not stable; people are not stable; people are rather a role that is constantly changing with the use of intelligent machines. This calculation example only considers the situation that there are 1 and 2 obstacles in the workspace, each database contains 180 sets of data, and the setting of the initial motion trajectory of the robot is the same as the previous one. The dynamic performance of the social role of the machine is that the process of social role formation is affected by other factors, such as the degree of the flow of human-computer interaction, the degree of human involvement in the interaction, and so on. These factors or conditions are flexible and uncontrollable. In addition, the effect of the social role that can be achieved varies depending on the degree of machine intelligence. The social role properties of machines are not invariable; a machine is a social role only when it interacts with and influences people; in other words, active and interacting machine intelligence is a machine social role. With the support of big data and the progress of deep algorithms and other technologies, human beings have entered the era of artificial intelligence. Modern human-robot interaction is based on machines running according to programs written in advance by humans. In essence, human intentions reflected in the behavior of all robots. Intelligent machines take certain actions based on preprogrammed instructions from human programmers. Even intelligent robots make certain decisions on their own, based on algorithmic judgment. However, with machines having dialogue and behavioral feedback systems that are highly close to humans, close to real people's natural language understanding and output and behavioral feedback, human-robot social interaction arises along with it. Intelligent robots are a completely new product born in recent decades, and human-machine relationships and human-machine interactions are also a completely new subject. The construction and understanding of human-machine social interactions are both to help robots integrate into human and social systems and to build systems for good human-machine interactions. Machines becoming social actors is a prerequisite for human-robot social interaction. Intelligent machines with dialogue and behavioral feedback systems that are highly like those of humans and capable of adaptive autonomous learning become social actors with certain norms and dynamically changing behavior patterns when they participate in human-robot social interaction as subjects.

### 3.2. Fuzzy Neural Network Algorithm Design

Designing a type-one fuzzy logic system involves making many choices for each segment. We need to choose the type of fuzzification, the form of the affiliation function, the parameters of the affiliation function, synthesis, implication, and the type of demulsified, etc. The different choices for each link represent the different mathematical methods involved. For example, the firing level of a rule *R*^*n*^ can be written in the following form if it is calculated using the minimum t-parametrization. Although the EFPF-PSO algorithm has a chance to obtain an optimized trajectory with a smaller fitness value, not every calculation will converge to the minimum fitness value, and its standard deviation is relatively large. However, the results obtained by the RPSOMP method are more stable, the standard deviation is small, and the convergence speed is faster.

(1) $$\begin{array}{c} \mu_{R^{n}}\left(x\right)=\arg\;\max\left\{\mu_{A_{i}^{n}}\left(x_{i}^{2}\right)\right\}. \end{array}$$

The output fuzzy set can be obtained by calculating the ignition level and the fuzzy rule posterior fuzzy set using the t-parametric operator. The final output fuzzy set B can be obtained by combining all the output fuzzy sets by t-residual van. To obtain the output clear value *y*, the output fuzzy set B needs to be defuzzied using a fuzzifier, and if the center of gravity fuzzifier is chosen, the output of the fuzzy logic system can be expressed as

(2) $$\begin{array}{c} y=\frac{\sum_{i=1}^{n}y_{i}\mu_{B}\left(y_{i}\right)}{\sum_{i=1}^{n}\mu_{B}{\left(y_{i}\right)}^{2}}. \end{array}$$

The high computational effort and complex design process of general type-two fuzzy logic systems constrain their application in control systems. In recent years, researchers in controller design have turned their attention to interval type-two fuzzy logic systems. The interval type-two fuzzy set A can be expressed as

(3) $$\begin{array}{c} A=\int_{x\in X}\int_{u\in J_{x}}\left(x,u\right). \end{array}$$

When *f*_*x*_(*μ*)=1, then the sub-subordinate function is an interval set, and if for both it holds, then the interval type-2 affiliation function (IT2MF, Interval Type-2 Membership Function) is obtained. The interval type-2 sub-subordinate function reflects the consistent uncertainty at the primary affiliation of *x*. Compared with the general type-two fuzzy logic system, the interval type-two fuzzy logic system greatly reduces the computational effort. Since interval type-two fuzzy sets also belong to type-two fuzzy sets, we can obtain

(4) $$\begin{array}{c} J_{x}=\left\{\mu \in \left[0,1\right]|\mu_{A}\left(x,u\right)=1\right\}. \end{array}$$

In this paper, the center of sets type reducer is used to calculate the reduced type of the obtained ignition output set. Since both the antecedent and the consequent parts are interval type-two fuzzy sets, the obtained reduced typeset is still an interval set. The procedure of degenerate calculation: firstly, the output space Y is discretized into an appropriate number of points, and the center of gravity of the interval type-II fuzzy set of the posterior is calculated by the following equation:

(5) $$\begin{array}{c} C_{B_{j}^{n}}=\frac{\int_{\theta \in J_{y1}}\int_{u\in J_{x}}\left(x,u\right)}{\sum_{i=1}^{n}y_{i}\mu_{B}\left(y_{i}^{2}\right)\sum_{i=1}^{n}\mu_{B}{\left(y_{i}^{2}\right)}^{2}}. \end{array}$$

To achieve the landing of the robot from the virtual prototype model to the real machine model, the first step that needs to be done is to draw a vector diagram for the real machine model. According to the size of the virtual prototype, the drawing size is marked on the vector diagram, and the commonly used drawing structure software for the drawing includes AutoCAD (Autodesk Computer-Aided Design) and CorelDRAW. In this article, we chose AutoCAD as the drawing tool. From equation (5), the center of gravity of the posterior interval type-II fuzzy set can be calculated in advance outside the control loop, which can reduce a large amount of computational burden for the control chip. The equation for the center-drop type of the set is

(6) $$\begin{array}{c} Y_{\cos}=\frac{\sum_{j=1}^{J}f^{j}}{\sum_{j=1}^{J}f^{j}y^{j}}. \end{array}$$

Therefore, to calculate the center of the interval set *Y*_cos_, only its left endpoint *y*_*l*_ and right endpoint *y*_*r*_ need to be calculated. The calculation of the left and right endpoints is given by the following equation:

(7) $$\begin{array}{c} y_{l}=\frac{\sum_{j=1}^{L}f^{j}y^{j}-\sum_{j=L+1}^{J}f^{j}y^{j}}{\sum_{j=1}^{R}f^{j}}, \end{array}$$

(8) $$\begin{array}{c} y_{r}=\frac{\sum_{j=1}^{L}f^{j}y^{j}-\sum_{j=L+1}^{R}f^{j}y^{j}}{\sum_{j=1}^{J}f^{j}}. \end{array}$$

The values of *L* and *R* in equations (7) and (8) are the values of the transition points needed to calculate the endpoint values, and the values of the left and right endpoints need to be calculated using the Karnik–Mendel (KM) algorithm. The design goal of the controller is to apply it to the trajectory tracking control of the Delta parallel robot and to provide a basis for subsequent research. The trajectory tracking control system of the Delta parallel robot uses three identical fuzzy logic controllers to control each of the robot's three servo motors. The demanded trajectory of the TCP is converted into the demanded angle of the drive joint by the kinematic inverse solution calculation and input to the demand trajectory of the TCP is converted into the demand angle of the drive joint by the kinematic inverse solution and input to the controller [20]. The input to the controller is the difference between the demand angle of the drive joint and the actual angle and its derivative. The T1FLC, which is the benchmark for the IT2FLC design, uses a type-one fuzzy set of trapezoidal affiliation functions as the antecedent and a type-one fuzzy set of triangular affiliation functions as the precedent.

**Figure 3:**
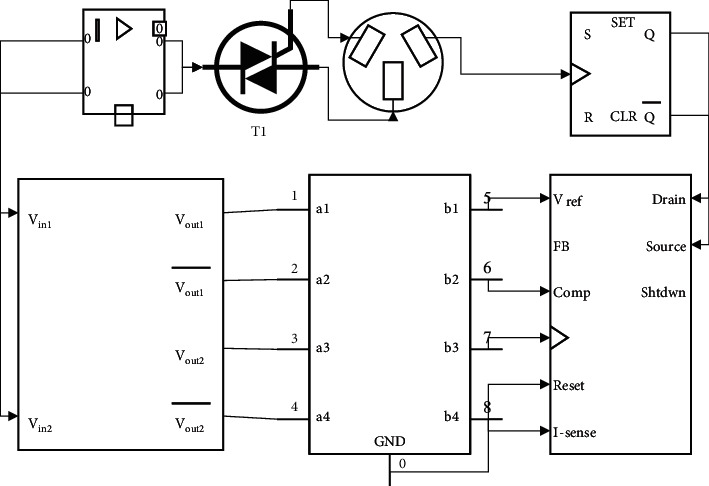
Fuzzy logic control system for Delta's robot.

The terms positive, negative, and zero used in the above fuzzy rules are expressed in the form of a type-one fuzzy affiliation function. The initial parameters of the type-one fuzzy set in T1FLC are empirically given artificially and adjusted to achieve a stable and continuous initial control performance of the robot system. The initial design of the input and output fuzzy sets applied to the trajectory tracking control of drive joint 1 is shown in Figure 3. The initial design structure of T1FLC for drive joint 2 and drive joint 3 is the same as that of drive joint 1.

Three types of IT2FLC are obtained using these three fuzzification methods, and these three types of IT2FLC are applied to the trajectory tracking control task of the Delta robot so that the TCP of the robot moves along the trajectory given by the following equation:

(9) $$\begin{array}{c} \left\{\begin{array}{c} x=0.1\;\cos\left(0.5\pi t+\pi \right)+0.2,\\ y=0.1\;\sin\left(0.5\pi t+\pi \right),\\ z=0.2547. \end{array}\right. \end{array}$$

**Figure 4:**
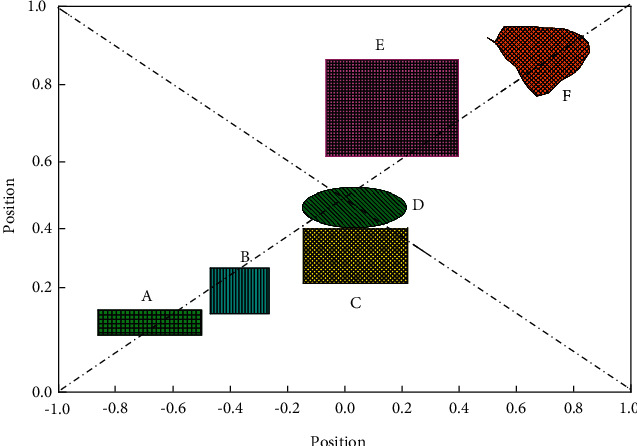
Trapezoidal affiliation function fuzzification method.

The fuzzy degree *α* ∈ [0,1], which takes a value every 0.1, can construct one IT2FLC for each fuzzy degree value, and five trajectory tracking control simulations are performed for each constructed IT2FLC to reduce the statistical error of the simulation results. The root means square error (RMSE) values of the three joints obtained from the simulation are plotted as shown in Figure 4. It can be observed from the figure that the RMSE value increases with the increase of fuzziness, and the RMSE value obtained by the two-end fuzzy method is larger than the other two methods. The reason for this phenomenon can be explained by Wu's work, when using the two-end fuzzy method, the FOU area produced by the same fuzzy degree is larger, and the larger FOU gives the fuzzy logic controller a larger damping characteristic. Robot control methods are divided into external command control and robot decision-making system automatic adjustment control. In the external command control, the robot needs to have a signal receiver that can communicate with the outside. The decision-making control requires the robot to be able to perceive the surrounding road conditions, audio, and video signal, etc. Increasing the damping characteristics of the controller can lead to more stable control performance, but it also causes a decrease in control accuracy. Therefore, the RMSE values obtained by using the two-terminal fuzzy method are larger than those obtained by the other two methods. The solution to the control accuracy degradation problem will be presented in the next section. In summary, since we need to obtain a controller with high nonlinear characteristics and smoother control performance, the IT2FLC obtained by the two-end fuzzy method is the structure of the type-two fuzzy logic controller that best meets the expectations of our control system characteristics.

The platform layer needs the driver layer and the algorithm layer to work together to complete the task. First, the platform layer receives the sensor signal of the driver layer. The quadruped robot has Bluetooth sensors and balance sensors on the body. The robot is always receiving multiple sensors during its movement. Signal, the robot system needs to fuse these sensor data and then realize the processing of different sensor data through the corresponding algorithm in the upper algorithm layer. The steering gear control interface is mainly to send the steering gear control signal to the actuator. This summary mainly tests the static gait walk state, as well as the dynamic gait trot state and backward motion and left-to-right turning motion of the robot dog proposed in this paper. The test environment was carried out on a smooth road to verify whether the motion pattern of the real machine and the motion gait based on Hop's oscillation model proposed in this paper are consistent, and the wake gait motion of the robot dog can be obtained through the video screenshots during the experiment. The robot dog can achieve a smooth and straight line, and the robot's walk state motion period *T* is 0.8 s, the step length of one cycle is 0.12 m, and the movement speed is 0.15 m/s through the test.

## 4. Analysis of Results

### 4.1. System Performance Results

**Figure 5:**
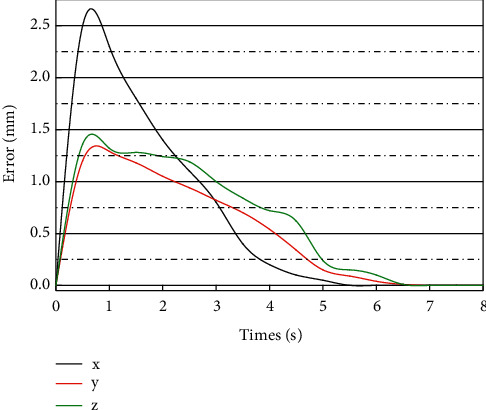
TCP position error using a controller.

A stable and effective interval type-II fuzzy logic control system can be designed using the dependent design method. Three simulation examples are used to investigate the control performance of the proposed SLIT2FNN controller. For comparative study, the conventional controller and the SLIT2FNN controller are connected separately to the Delta robot for trajectory tracking simulation experiments. The control goal is to make the TCP of the Delta parallel robot move along the given trajectory with as low as possible position and velocity errors and achieve high trajectory tracking accuracy regardless of the internal and external uncertainties. The RMSE values of the angles of the three drive joints and the positions of the TCP in the workspace were recorded as the indexes for evaluating the control performance after the TCP of the robot ran along the trajectory given by equation (9) for one working cycle (8 seconds). The trajectory tracking errors of the drive joints and the TCP of the SLIT2FNN controller and the conventional PD controller are shown in Figure 5.

As we can observe in Figure 5, at the beginning of the simulation (about 1.7 s), the SLIT2FNN controller is in the process of learning the environmental dynamics, and the neural network adjusts its structural parameter values through the parameter self-learning algorithm, while the control signal is mainly provided by the PD controller in the parallel control structure. At the end of the neural network self-learning process, the IT2FNN replaces the conventional PD controller as the main control signal output in the parallel control structure, while the tracking accuracy of the robot is significantly improved as can be seen in the figure. The social interaction between man and machine will follow. Intelligent robots are a completely new product born in recent decades. Human-machine relationship and human-computer interaction are also a brand-new subject. The construction and understanding of human-computer social interaction is to help robots integrate into human and social systems.

**Figure 6:**
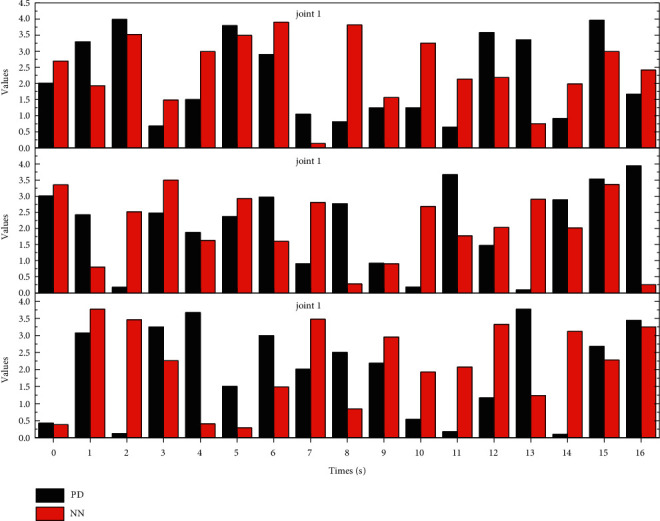
Control signals.

A second simulation experiment is given to demonstrate more intuitively the learning process of the SLIT2FNN controller and the effect of parameter self-learning on the control performance. In this simulation, the same robot trajectory is used as in the first simulation, but the robot is required to run along this trajectory for two consecutive working cycles. In the first work cycle (8 seconds), only the conventional PD controller is involved in controlling the trajectory tracking of the robot. In the second work cycle, IT2FNN is connected in parallel to both sides of the PD controller to form a parallel control structure to complete the remaining trajectory tracking process.  Figure 6 gives the output signals of IT2FNN and PD controller during this simulation experiment. It can be observed that when the IT2FNN is connected in parallel to both sides of the PD controller, the structural parameters of the IT2FNN start the self-learning process, and after the learning time of about 1.7 seconds, the output signal of the IT2FNN replaces the PD controller as the main output source of the control signal, which is the same as the result of Simulation 1. The above simulation results demonstrate the effect of the learning process of SLIT2FNN on the controller output and the improvement of the control accuracy in the second operating cycle with the parallel intelligent control structure.

It is important to note that the SLIT2FNN controller proposed in this chapter does not require any information about the mathematical model of the controlled object. In practice, there are a variety of intrinsic and extrinsic uncertainty effects, and if the PD controller used by the robot is tuned for one operating environment to achieve optimal control, it does not mean that the same control quality can be obtained in other operating environments. It is impractical to perform optimal adjustment of PD control parameters for each case. Therefore, the parallel control system structure with self-learning capability proposed in this paper is of more practical value. This controller with parallel structure can not only provide the control effect of the corresponding PD controller when the neural network learns, but also make the control accuracy and robustness improvements to a great extent after the learning is completed and have higher environmental adaptability. At the same time, the parallel control structure proposed in this paper makes it easy to be applied to existing automation equipment and can greatly save hardware upgrade costs.

### 4.2. Neural Network Motion Results Analysis

**Figure 7:**
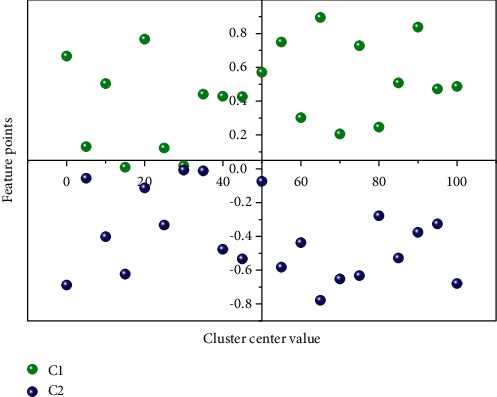
Clustering center of the first feature layer of EFPFDB.

To verify the optimized trajectory reuse algorithm, this section constructs the optimized trajectory database generated by the EFPF-PSO algorithm into a data index structure with two levels and six-leaf nodes using a vocabulary tree. Due to the limitation of the computer's computing power and time relationship, this algorithm only considers the case of 1 and 2 obstacles in the workspace, each database contains 180 sets of data, and the initial motion trajectory of the robot is set the same as before. This algorithm can generate corresponding optimized trajectory databases for specific application scenarios in practical applications and index the databases according to the actual situation by classification. Firstly, the EFPFDB of the optimized trajectory is clustered for the first time by K-mean clustering algorithm; considering the characteristics of the number of obstacles, here the classification value *K* = 2. The clustering centers of the first feature layer of the database are C1 and C2, as shown in Figure 7. The algorithm has obtained the relationship between the location features of obstacles by unsupervised learning, and the EFPF values are classified.

**Figure 8:**
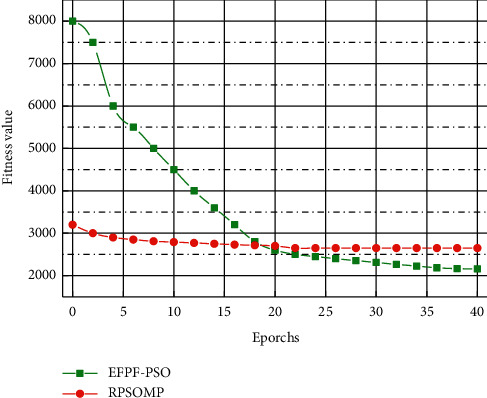
Average of the adaptation of RPSOMP and EFPF-PSO.

The RPSOMP method consists of a PSO-based motion trajectory optimization algorithm and an optimized trajectory reuse method. The input is the trajectory to be optimized, and its EFPF vector is calculated first, and the optimized trajectory adjustment with the highest similarity EFPF stored in the database is calculated by vocabulary tree query. The preliminary optimized trajectory after the query is obtained using equation (9), and if the obtained trajectory is a feasible solution, the obtained trajectory is directly used for motion planning as the output result. This paper uses the center of sets type reducer to reduce the type of the ignition output set obtained. Since the antecedents and consequents used in this paper are both interval type-two fuzzy sets, the reduced-type set obtained is still an interval set. If the obtained optimized trajectory is not a feasible solution, the optimized trajectory given by the algorithm is used to initialize the PSO particles. The adjustment amount of the query result trajectory is used as the mean value of the particle initialization distribution, and the value calculated from the query result is used as the standard deviation to initialize the particle positions of the PSO and perform the optimization calculation. Finally, the adjustment amount of the obtained optimized trajectory and the EFPF of its initial trajectory are stored in the corresponding database, and the optimized trajectory is output. The method flowchart of the RPSOMP method is shown in Figure 8.

From the analysis in Figure 8, RPSOMP converges faster than EFPF-PSO, taking only 0.43 seconds on average, which fully satisfies the requirements for trajectory optimization calculation in engineering applications. Meanwhile, it should be emphasized that, in practical applications, if the data volume of the optimization database is large and the database is designed for a specific problem, in most cases, the RPSOMP method does not require repetitive optimization calculations but can directly use the retrieval results to generate optimization trajectories. This can further improve the time to obtain the optimized trajectory to about 0.001 seconds and meet the demand of real-time trajectory optimization. Although the EFPF-PSO algorithm has a chance to obtain optimized trajectories with smaller fitness values, not every calculation converges to the minimum fitness value, and its standard deviation is relatively large. In contrast, the results obtained using the RPSOMP method are more stable, have smaller standard deviations, and converge faster. For the robot online collision detection problem, a simplified characterization method of robot and obstacle in space is proposed. Based on this, the robot trajectory optimization problem is investigated using the PSO-based motion trajectory optimization algorithm. The demand trajectory of TCP is converted into the demand angle of driving joints and input into the controller through the inverse kinematics calculation. The input of the controller is the difference between the required angle of the drive joint and the actual angle and its derivative. As the IT2FLC design benchmark, T1FLC uses a type 1 fuzzy set of trapezoidal membership function as the antecedent and a type 1 fuzzy set of triangular membership function as the subsequent piece.

## 5. Conclusion

This method is based on the optimized T1FLC, and the fuzzy degree is used to fuzzify the type-one fuzzy subordinate function into the interval type-two fuzzy subordinate function to obtain the IT2FLC. Three fuzzification methods for the trapezoidal subordinate function are studied, and the relationship between the fuzzy degree, the fuzzification method, and the output control surface is given. OSEC is used to ensure the control accuracy of IT2FLC after fuzzification, and the calculation and value of OSEC are given. A SLIT2FNN with a seven-layer structure is established, and a parallel intelligent controller based on SLIT2FNN is proposed on this basis. The transient performance during the learning process of the neural network can be compensated by the parallel PD controller, which makes it easy to upgrade the conventional controller. In the design of the front piece set of SLIT2FNN, the dual sequence symmetric trapezoidal affiliation function arrangement method is used to make the system's self-learning law and stability analysis in analytic form. In the design of the neural network self-learning law, a parameter self-learning algorithm based on the SMC theory is established to adjust the structural parameters of IT2FNN online. An industrial intelligent robot software system based on the robot operating system architecture is established, and the kinematic and dynamic models, mechanism parameter optimization algorithms, SLIT2FNN-based intelligent controller, and RPSOMP method are implemented based on this paper. Based on the research in this paper, the intelligent task planning module is further investigated to make the robot autonomous and capable of operating in unstructured spaces and environments and completing nondeterministic tasks online in real time.

## Data Availability

The data used to support the findings of this study are available from the corresponding author upon request.
